# Demand-side financing: can it help deliver eye care for all?

**Published:** 2022-09-20

**Authors:** BR Shamanna, GVS Murthy, Thulsiraj Ravilla, Iain Jones

**Affiliations:** School of Medical Sciences, University of Hyderabad, Hyderabad, India.; Director – Indian Institute of Public Health, Hyderabad, India.; Executive Director: Lions Aravind Institute of Community Ophthalmology, Aravind Eye Care System, Madurai, India.; Senior Global Technical Lead Economics Research: Sightsavers, UK.

**Demand-side financing mechanisms in eye care can be a tool to achieve universal eye health coverage by increasing access and utilisation of key eye health services.**

Many people in low- and middle-income countries have inadequate access to affordable eye care.^1^ This is, in part, due to the way eye care services are financed – financing arrangements influence what services are provided, where services are available, who has access to them, and how people pay for them.

**Figure:**
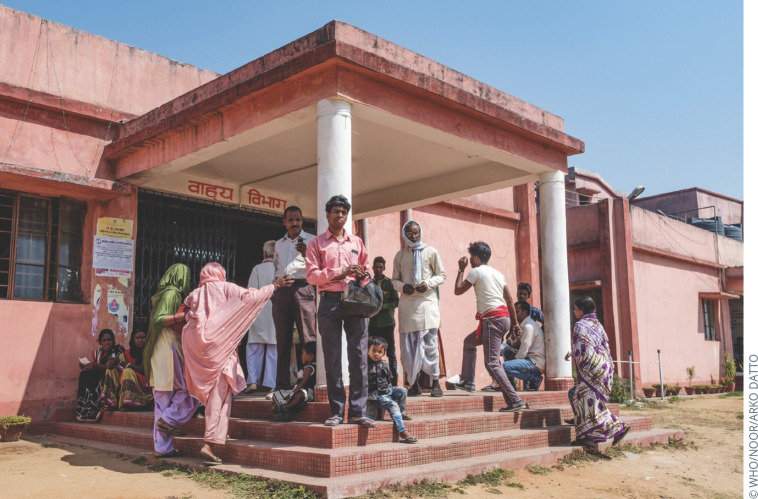
Exterior of the Sadar Hospital, Ramgarh. india

Most health care financing in low- and middle-income countries supports the supply of services, channelling payments to service providers. For example, governments allocate budget to hospitals to ensure they provide services to address population health needs. This is known as supply-side financing.

However, insufficient or poorly allocated supply-side financing can lead to inadequate and unequal distribution of certain health services. This can result in poor quality of care and service provision focused on urban populations, leaving many people in rural areas with limited access to affordable care. However, efforts to achieve financial risk protection and to improve access and quality of service provision for all have fostered different financing arrangements – demand-side financing mechanisms.

## Demand-side financing

The World Health Organization's World Report on Vision noted that the use of eye care services is determined by the availability, accessibility, affordability, and acceptability of such services.^1^ Barriers, including the actual and perceived direct and indirect cost of eye care, can mean that people do not access and use eye health services. Demand-side financing (see panel) provides a way of channeling scarce resources in a way that increases the use of health services amongst specific groups, especially those that cannot afford them. The core features of this type of financing are^2^: a pre-specified target group, such as poor households; financial transfers to the beneficiaries, such as vouchers or conditional cash transfers; and a rationale for the choice of services covered, such as services with limited supply or services that are not demanded due to market failures.

**Demand-side financing** is a model that makes health funding or subsidies from various sources, including governments, directly available to people in need, so they can afford, and pay for, health services. The World Health Organization defines it as a tool to “improve the uptake of under-used services […] by placing purchasing power, as well as the choice of provider (where possible), directly in the hands of the recipients.”^2^

Demand-side financing offsets part, or all, of the cost of a health service by providing funds or subsidies directly to people to increase their purchasing power. People are given direct access to a health care ‘budget’ that allows them to choose, within predetermined limits, from which providers they would like to purchase health services (e.g., cataract surgery).

An example of this is the Pradhan Mantri Jan Arogya Yojana national health protection scheme launched in India in 2018 (see panel), which was adopted across the country with some local modifications. Each family is given a ‘health card’, which can be used to pay for services, with some boundaries on procedures and providers. Initial information available from the information portal has indicated an increase in people's access to care, particularly surgical procedures. It puts the power of ‘purchasing health’ in the hands of the buyer; meaning that people can get their health care from where they want and when they want it. It is administered under an insurance mechanism to reduce misuse and make it easier to manage efficiently.

There are several examples of demand-side financing in the health sector, including cash transfers and voucher schemes^3^ to help tackle malaria^4^ and improve maternal health.^5^,^6^ The lessons from these initiatives are as follows.

- Demand-side financing empowers consumers by increasing their purchasing power; there has been increased use of services in many schemes.
- It encourages innovation.
- There is a better use of resources and improved efficiency of service providers.
- There is the positive impact of involving community health workers, working with local communities to identify people most in need, raising community awareness, and fostering community ownership.

**Pradhan Mantri Jan Arogya Yojana: India's national health protection scheme**

The Pradhan Mantri Jan Arogya Yojana, popularly known as Ayushman Bharat, launched on 25 September 2018 and is the national health protection scheme (NHPS). It aims to secure the lives of 500 million individuals (or 107.4 million low-income rural and urban households), with a defined benefit cover of Rs 500,000 (roughly US $6,286) per family. NHPS is a demand-side financed national public health insurance fund that aims to provide free access to health through insurance coverage, paid for by the government. This would cover roughly half of the population in the lower economic strata. Several states have launched a similar programme as a state initiative. The aim is to protect the economically vulnerable populations from catastrophic health expenses that can financially ruin them.

Under this scheme a large number of surgical procedures are covered, including hospitalisation. Many of the state health protection schemes do not cover cataract surgeries, as they are already provided free under the National programme for Control of Blindness and Visual Impairment. It does cover a wide range of interventions – retinal surgeries, glaucoma, squint correction, corneal transplants, etc. Those covered under this scheme get such surgical interventions at no cost from government hospitals, accredited private hospitals, and not-for-profit hospitals. A supply-side component to strengthen primary health, including eye care services, includes the establishment of health and wellness centres for predominantly preventive, promotive and initial curative services at an out-patient level, offered free of cost.

## Limitations

Demand-side financing also has its limitations. Initiatives seem to work best when there is an established supply of service providers and enough health workers. Demand-side financing may not be sustainable in remote areas where service provision is limited unless there is significant, complementary supply-side investment. In many parts of the world, people live far away from hospitals and simply getting to the nearest health provider is a challenge. People in these circumstances do not have access to a range of hospitals or health care providers to choose from. In these communities, investing to improve the quality of the health systems and delivery of primary eye health care services would be needed before an effort is made to encourage the use of services through demand-side financing.

## Opportunities for demand-side financing in eye care

An early example of demand-side financing in eye care in a low- and middle-income country is an initiative in Bangladesh. A voucher scheme for cataract surgery in a district programme was introduced in the mid-2000s, but it is yet to be part of the national programme.^7^ The programme conceptualised and provided opportunities for people who needed cataract surgery to use a range of providers (public and private) for their surgery on redemption of a voucher.

So, what should be the role of demand-side financing in increasing the accessibility and affordability of eye care services? There is no doubt that these mechanisms are tools that could be used to achieve universal eye health coverage by increasing access and utilisation of key eye health services. However, supply-side issues and sustainability challenges need to be thought through carefully before implementing demand-side financing initiatives. There is a need to generate more evidence on the impact, costs, cost-effectiveness, and equity implications of these initiatives to ensure the best use of limited resources. This knowledge will help define when, where, and how demand-side mechanisms could help in delivering integrated, people-centred eye care and making eye care affordable to all.
